# Supplementary material for: Evaluation of Food Fineness by the Bionic Tongue Distributed Mechanical Testing Device
Source: Sensors (Basel). 2018 Dec 3;18(12):4250. doi: 10.3390/s18124250 (PMC6308582; doi:10.3390/s18124250)
Supplement: Supplementary file 1 [file sensors-18-04250-s001.pdf]

Supporting Information:

In this paper, the constructed **bionic tongue distributed mechanical testing device** was used to conduct the **contact pressure experiments on foods**, and the **food fineness perception evaluation model** was established based on the obtained pressure data, which is to **explore the fineness texture perception among the different experimental samples.** In terms of the experimental materials, according to the comments gave by the reviewers, we respectively selected three kinds of cookie samples among WZ Cookie, ZL Cookie and JSL Cookie (**the same kind of food**), and three kinds of different foods among the apple, banana and cookie (**different kinds of food**), to be as the experimental samples. In the manuscript, we only explored the fineness perception of the same kind of food sample. **Next, we will supplement the study of the fineness perception between the different kinds of food samples.**

1. Experimental Section

1.1. Experimental Material

According to the classification methods of mechanical properties about food texture, we referred to GB/T 29604-2013 Sensory Analysis-General Guidelines for Establishing a Reference for Sensory Characteristics [1] and selected three kinds of solid foods with different texture characteristics such as apples, bananas and cookies as experimental materials. Wash and peel the apple, then use a knife to cut the sheet with a thickness of 10mm along the longitudinal axis. The apple sample was prepared using a 10 mm diameter sample preparation device to form a cylinder with a diameter of 10 mm and having 10 mm height. Wash and peel the banana, then use a knife to cut the sheet with a thickness of 10mm. The banana sample was also made by using a 10 mm diameter sample preparation device to form a cylinder with a diameter of 10 mm and having 10 mm height. The cookie was cut with a knife into a cube with the side length of 10 mm, and the sample without cracks was taken. After each test, the bionic tongue indenter and the stage were cleaned to ensure the consistency of measurement conditions.

1.2. Sensory Assessment

The food sensory assessment is a kind of statistical-based psychological activity, which is a quantitative and qualitative description of the food by consumers through sensory perception. According to the national standard GB/T 10220-2012 Sensory analysis—Methodology—General guidance [2]. 20 evaluators (10 men and 10 women) were selected and trained to constitute in an evaluation group to conduct sensory assessment on sample texture of apple, banana and cookie. Under the precondition of guaranteeing sample texture features and basic sampling principles, the samples were numbered and the samples are numbered and submitted to the evaluator for independent evaluation according to a random coding. What's more, we should guarantee the evaluator to wear an eye mask to avoid subjective psychological cues due to its appearance and color. The taste detergent was used to remove residual taste in the oral cavity between two samples or two evaluation activities. Moreover, we used a linear scale detection method to mark the intensity of the sensory perception on a linear scale of 10 cm. The two endpoints of the 10cm line correspond to the lowest score and highest score of the sensory assessment. Each sample was evaluated for three times and it was summarized by using the mean value method. In the end, the linear scale was converted into the corresponding

value in proportion for statistical analysis.

2. Results and Analysis

2.1. Contact Pressure Distribution

In this experiment, there were three kinds of food including apple, banana and cookie. Each food had 3 parallel samples. The device in the Figure 10 was used for contact pressure test. According to the above experimental method, each parallel sample was tested under 8 bionic tongue indenters with different curvature differences. In the data collection interface of the analysis software, the image of pressure distribution at each moment in the sensing region of the arrayed film pressure sensor in constant speed pushing process was displayed in real time. The image was featured by the annular distribution with the center of peak stress. The image of pressure distribution and the matrix of the pressure values corresponding image at each moment was stored in the data frame. We set to acquire one data frame every 0.02s in this experiment.

The data frame covered from contacting different foods to leave food pieces completely was different. According to the characteristics of the experimental data among the three foods, the maximum range in the data frame was selected as the subsequent calculated sample size, that was, the 17-80 data frames were defined as valid data frame. Within the valid data frame, the maximum matrix of pressure value was 14×12. Thus, the middle sensing area 5×5cm^2 of the arrayed film pressure sensor was defined as the valid pressure collection area.

Considering that the curvature distribution of 8 bionic tongue indenters' surface has a large difference, which results that mechanical information perceived by different bionic tongue indenters are different. The images corresponding to the maximum total pressure in the apple sample perceived by 8 bionic tongue indenters were shown in Figure 1.

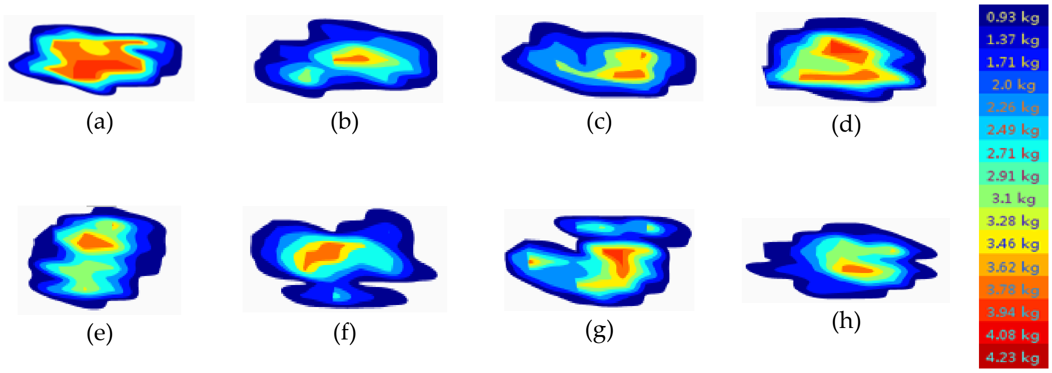

Figure 1. The image corresponding the maximum pressure of the apple sample perceived by 8 bionic tongue indenters. The difference in color in the image indicates the difference in pressure data, and the color gradually transforms from blue to red, representing the change in pressure from small to large: (**a**) S1; (**b**) S2; (**c**) S3; (**d**) S4; (**e**) S5; (**f**) S6; (**g**) S7; (**h**) S8.

From figure 1, it can be seen that the bionic tongue distributed mechanical force testing device can obtain a large amount of experimental data. The pressure matrix over the contact surface in each data frame during the pressing contact process can lay the foundation for the study of food fineness.

2.2. Establishment of Fineness Perception Evaluation Model

In this study, we used the distributed pressure data from the valid data frame of the contact pressure distribution map to establish a food fineness perception evaluation model. According to the pressure distribution change data, we selected 3 major indicators to establish the food fineness perception evaluation model. Three indicators have been introduced in the manuscript.

Three indicators including gradient, stress change rate and areal density are the same as the index to describe the fineness, which are weighted to get the comprehensive fineness index *I*:

$$I = \alpha G_{min} + \beta M_{min} + \gamma \rho_{min} \tag{1}$$

Where α, β, γ are the weight coefficient of gradient value, stress change rate, areal density.

In order to avoid the influence of subjective randomness, this paper uses entropy weight method to determine the weight objectively for three indicators including gradient, stress change rate, areal density. The entropy weight method uses the information entropy to calculate the entropy of each index based on the degree of variation of each index. And then, the weight of each index is corrected by the entropy weight, and the objective weights of three indexes are obtained. Proceed as follows:

1) Data Standardization

The matrix $I = (i_{uv})_{st}$, i_{uv} represents the value corresponding to the vth index of the u-th evaluation object (u=1,2, ··· ,72, corresponding to three kinds of food including apples, bananas and cookies under 8 bionic tongue indenters, three samples are measured for each food under each bionic tongue indenter. v =1, 2, 3, corresponding to three indicators including gradient values, stress change rates and areal density). The matrix is represented as:

$$I = \begin{bmatrix} I_{11} & I_{12} & I_{13} \\ I_{21} & I_{22} & I_{23} \\ \cdots & \cdots & \cdots \\ I_{u1} & I_{u2} & I_{u3} \end{bmatrix}_{72\times3} \tag{2}$$

Standardized formula is represented as:

$$l_{uv} = \frac{i_{uv} - min(i_{u\cdot})}{max(i_{u\cdot}) - min(i_{u\cdot})} \tag{3}$$

The standardization matrix $L = (l_{uv})_{st}$ is gained by calculating, expressed as:

$$L = \begin{bmatrix} L_{11} & L_{12} & L_{13} \\ L_{21} & L_{22} & L_{23} \\ \cdots & \cdots & \cdots \\ L_{u1} & L_{u2} & L_{u3} \end{bmatrix}_{72\times3} \tag{4}$$

2) Index Information Entropy Acquisition

The information entropy Ev of the v-th indicator can be expressed as:

$$E_v = -\frac{1}{\ln(s)} \sum_{u=1}^{s} p_{uv} \ln(p_{uv}) \tag{5}$$

where, $p_{uv} = l_{uv} / \sum_{u=1}^{s} l_{uv}$, when p_{uv}=0, then the following equation is established as:

$$\lim(p_{uv}lnp_{uv}) = 0 \tag{6}$$

If the entropy value Ev of a certain index is smaller, illustrating that the degree of variation of the index value is greater, the more amount of information provided, the greater the role of the index in comprehensive evaluation, and the greater the weight should be.

3) Weight of Each Indicator Acquisition

The weight expression of the vth indicator is as follows:

$$w_v = \frac{1 - E_v}{\sum_{v=1}^{t}(1 - E_v)} \tag{7}$$

The result of the calculation was expressed as: w1=0.339; w2=0.412; w3=0.249, that was, the weight coefficients of three indicators including gradient, stress change rate, areal density were equal to 0.339, 0.412, 0.249.

The indicator weight values obtained were brought into the model formula (1). According to the food fineness perception evaluation model established based on sensing mechanical information on the bionic tongue indenter surface, the fineness index values under the different bionic tongue indenters and the different kinds of food samples were calculated. The following figure shows the average of the fineness index values of the three kinds of foods in steady status under 8 bionic tongue indenters, as shown in Figure 2.

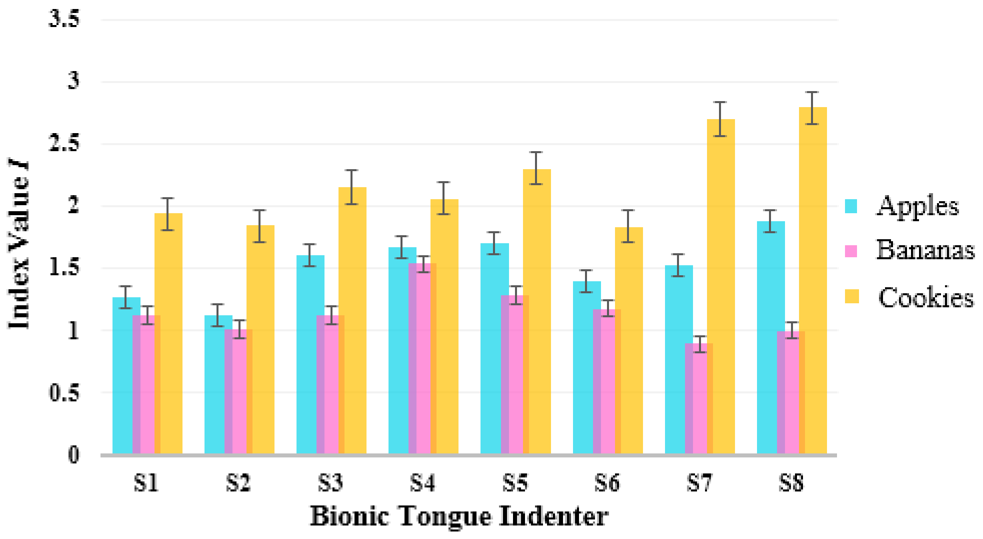

Figure 2. The average of fineness index values about three kinds of foods calculated by the fineness perception evaluation model based on the mechanical information perceived by 8 bionic tongue indenters.

According to the fineness index values obtained from the model, the fineness index values of three kinds of foods were plotted to fit the stress-fineness curve under 8 different indenters. The following figure shows only the fitted stress-fineness curve of the three kinds of foods under the bionic tongue indenter S2, as shown in Figure 3.

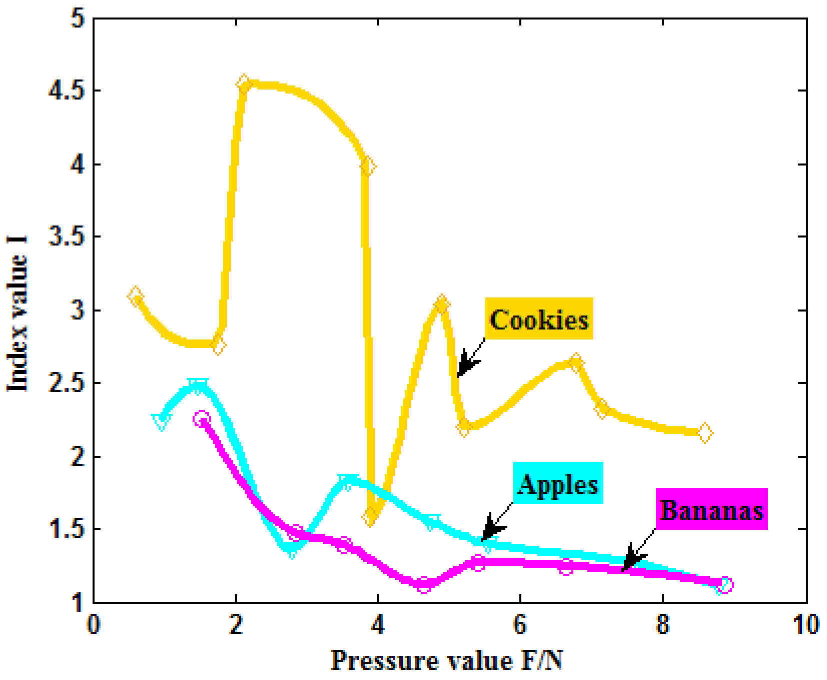

Figure 3. Stress-fineness curves of three kinds of foods under the bionic tongue indenter S2.

Figure 3 shows the changing process of the fineness index value, which was following the gradually increasing pressure on apples, bananas and cookies by using bionic tongue indenter S2. According to the defined three indicators including gradient, stress change rate and areal density, the smaller the gradient index value is, the finer the food pieces in the frame is; the smaller the stress change rate, the finer the stress sensing point over tongue surface perceived; The smaller the areal density, the larger the contact area, indicating that the food pieces are finer. Therefore, the smaller the fineness index value is, which indicates that food pieces are finer. From the trend of the three curves, it could be seen that as the stress on the food block increased, the fineness index gradually decreased and tended to be stable, indicating that following the increasing pressure on the food block based on bionic tongue indenter device, the tongue surface could perceive that food pieces were more and more fine, and the fineness index tended to a stable value eventually. In the trend of three foods' fineness changing, it could be seen that the fineness of bananas is the best and the fineness of cookies is the worst, which is in line with the actual perception of human tongue.

2.3. Sensory Assessment

The apples, bananas, and cookies were evaluated by 20 evaluators for sensory assessment. The result was recorded on a straight line of 10cm. We measured it and made data statistics, among them, the two endpoints of the 10 cm line were respectively corresponding to the lowest score and the highest score of the sensory assessment, that is, the higher the score, the finer the perception. The fineness perception score results of three kinds of foods are as in Figure 4.

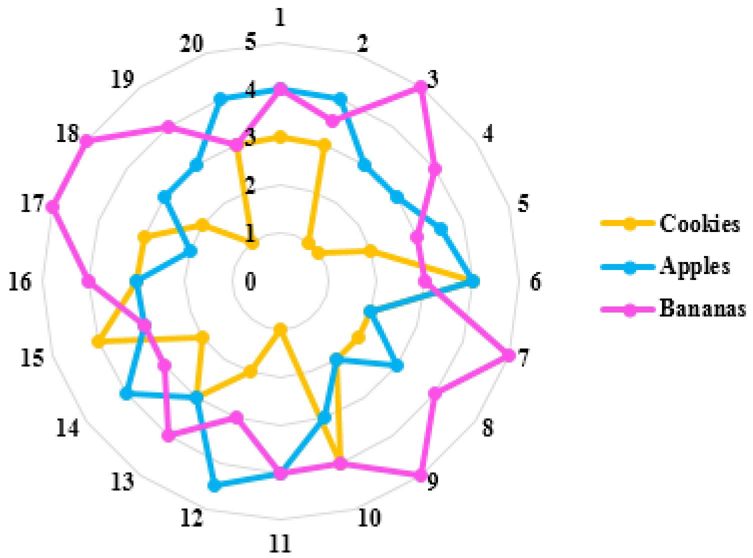

Figure 4. A radar diagram of the different evaluators to the fineness perception of three kinds of foods.

Figure 4 shows the sensory scores of the three kinds of foods by 20 evaluators. It can be seen that the fineness sensory scores of bananas are the highest. cookies have a lower score. It shows that the true fineness perception of humanity is that the fineness of bananas is the best, followed by the apples, and the fineness of cookies is the worst. The conclusion of the sensory assessment and the results from above-mentioned experiment are the same.

2.4. Correlation Analysis between Sensory Assessment and Fineness Evaluation Model Results

In order to explore the correlation between the food fineness perception evaluation model results established based on the mechanical information perceived by the bionic tongue indenter and the sensory assessment. Pearson correlation analysis was used to calculate the correlation coefficient and to obtain the correlation coefficient matrix. The correlation coefficient between the two variables X, Y is defined as:

$$\rho_{XY} = \frac{Cov(X,Y)}{\sqrt{Var(X)}\sqrt{Var(Y)}} = \frac{E[(X-EX)(Y-EY)]}{\sqrt{E(X-EX)^2}\sqrt{E(Y-EY)^2}} \quad (8)$$

where X is the sensory score of food fineness. Y is the fineness index calculated by the evaluation model. $Cov(X,Y)$ is the covariance of variable X and Y. EX is the mean of X. EY is the mean of Y. Var(X) is variance of X. Var(Y) is the variance of Y.

The sample correlation coefficient is expressed as:

$$r = \frac{\sum_{i=1}^{n}(x_i - \bar{x})(y_i - \bar{y})}{\sum_{i=1}^{n}(x_i - \bar{x})\sum_{i=1}^{n}(y_i - \bar{y})^2} \quad (9)$$

where x_i is a random sample of capacity n taken from variable X. y_i is a random sample of capacity n taken from variable Y.

Table 1. The correlation coefficient between the sensory results and the fineness indexes value of the 8 bionic tongue indenters.

experimental material	Bionic tongue indenters							
	S1	S2	S3	S4	S5	S6	S7	S8
Apples	0.827	1.000**	-0.979*	-0.932	0.890	0.732	-0.922	0.994**
Bananas	0.978*	-0.790	-0.743	0.981*	-0.710	0.999**	-0.998**	-0.859
Cookies	-0.923	-0.940	-0.993**	-0.943	0.982*	-0.984*	-0.813	0.838

Note：①* indicates that correlation is significant at the 0.05 level.
②** indicates that correlation is extremely significant at the 0.01 level.

Table 1 shows that we gained the correlation coefficients of three kinds of foods between the sensory assessment and fineness index values under the 8 bionic tongue indenters, and the average values of the apple's correlation coefficients is 0.910, the average values of the banana's correlation coefficients is 0.882, the average values of the cookie's correlation coefficients is 0.927, which are significantly correlated. Among them, three bionic tongue indenters including the end of Tiled Tongue S2, the end of Raised Tongue S6, the front of Overturned Tongue S7 have the fineness sense of apples and bananas. And the fineness perception results have extremely significant correlations with sensory assessment.

This shows that the food fineness perception evaluation model is effective, and the bionic tongue distributed mechanical testing device has a good practical significance for simulating to the food texture.

References

1. Zhao, L.; Zhi, R.; Liu, W. Sensory Analysis-General Guidelines for Establishing a Reference for Sensory Characteristics. *CHINA ZHIJIAN PUBLISHING HOUSE*, China, 2013; pp. 23-49.
2. Zhang, L.; Liu, J. Sensory analysis—Methodology—General guidance. *SAC*, 2012; pp. 7-19.
